# PHILOS Synthesis for Proximal Humerus Fractures Has High Complications and Reintervention Rates: A Systematic Review and Meta-Analysis

**DOI:** 10.3390/life12020311

**Published:** 2022-02-19

**Authors:** Lorenzo Massimo Oldrini, Pietro Feltri, Jacopo Albanese, Francesco Marbach, Giuseppe Filardo, Christian Candrian

**Affiliations:** 1 Service of Orthopaedics and Traumatology, Department of Surgery, EOC, 6900 Lugano, Switzerland; lorenzomassimo.oldrini@eoc.ch (L.M.O.); jacopo.albanese@eoc.ch (J.A.); francesco.marbach@eoc.ch (F.M.); giuseppe.filardo@eoc.ch (G.F.); christian.candrian@eoc.ch (C.C.); 2 Faculty of Biomedical Sciences, Università della Svizzera Italiana, Via Buffi 13, 6900 Lugano, Switzerland

**Keywords:** PHILOS, locking plate, proximal humeral fracture, PHF, osteosynthesis

## Abstract

Purpose: The aim of this study was to quantify the rate of complications and reinterventions in patients treated with PHILOS plate for proximal humerus fractures (PHFs) synthesis. Methods: A comprehensive literature search was performed on the PubMed, Web of Science, Embase, and Cochrane databases up to 7 October 2021. Studies describing medium and long-term complications in PHF synthesis using the PHILOS plate were included. A systematic review and meta-analysis were performed on complications and causes of reinterventions. Assessment of risk of bias and quality of evidence was performed with the Downs and Black’s “Checklist for Measuring Quality”. Results: Seventy-six studies including 4200 patients met the inclusion criteria. The complication rate was 23.8%, and the main cause was screw cut-out (4.1%), followed by avascular necrosis (AVN) (3.1%) and subacromial impingement (1.5%). In patients over 55 years, the complication rate was 29.5%. In the deltopectoral (DP) approach the complication rate was 23.8%, and in the delto-split (DS) it was 17.5%, but no difference between the two approaches was seen when considering the type of fracture. The overall reintervention rate was 10.5% in the overall population and 19.0% in older patients. Conclusions: Proximal humerus synthesis with a PHILOS plate has high complications and reintervention rates. The most frequent complication was screw cut-out, followed by humeral head AVN and subacromial impingement. These results need to be further investigated to better understand both the type of patient and fracture that is more at risk of complications and reintervention and to compare pros and cons of the PHILOS plate with respect to the other solutions to manage PHFs.

## 1. Introduction

Proximal humerus fractures (PHFs) represent 5% to 10% of all fractures, being the third most frequent in the elderly population after femur and wrist fractures [1], and these numbers are constantly increasing due to the aging of the population [2,3]. The most appropriate treatment should be chosen based on the patient’s age, bone quality, co-morbidities, compliance, and functional demands. Patients are frequently treated non-operatively, but when surgical treatment is necessary, different techniques can be performed such as percutaneous pinning, plating, arthroplasty, or intramedullary nail [4,5,6]. Among the surgical treatments, open reduction and internal fixation (ORIF) is the most used, although it presents several downsides such as an increased risk of avascular necrosis (AVN) of the humeral head, non-union, malunion, and screw cut-out [7].

To overcome these problems and increase patient functional outcomes, an anatomical plate design was developed [6,8,9] by the AO/ASIF group: the Proximal Humeral Internal Locking System (PHILOS) plate. The PHILOS plate is an internal fixation system that enables stabilization thanks to multiple angular stable interlocking screws, with the goal of preserving the biological integrity of the humeral head while securing an anatomical reduction [10]. This method of fixation allows early mobilization, and thanks to the presence of numerous holes in its proximal portion, it also allows if needed an anchorage for rotator cuff sutures. The indications for the use of the PHILOS plate are various: two-, three-, four-fragment dislocations of proximal humerus fracture including fractures in osteoporotic patients, pseudoarthrosis, and osteotomy in the proximal humerus [11]. The reliability of this device led the PHILOS plate to become the standard surgical treatment for the fixation of PHFs. Despite its large use, the focus has been largely placed on the functional results, while less attention has been paid to the rate and type of complications of this treatment approach [12].

The aim of this systematic review and meta-analysis was to quantify and critically analyze the rate of complications and reinterventions following surgical treatment with the PHILOS plate of PHFs.

## 2. Materials and Methods

### 2.1. Literature Search

A review protocol was developed based on the Preferred Reporting Items for Systematic Reviews (PRISMA) statement (www.prisma-statement.org, accessed on 1 October 2021). A comprehensive search of the literature was performed in the bibliographic databases PubMed, Web of Science, Embase, and Wiley Cochrane Library from inception up to 7 October 2021. The following research terms were used: ((proximal humer* OR shoulder OR humer*) AND (fractur*) AND (internal locking system OR PHILOS OR plate)). Comparative and non-comparative studies describing medium and long-term complications in PHF synthesis using the Synthes PHILOS^®^ plate were included. Only articles with a follow-up of more than 12 months were considered. Case reports or case series describing ≤ five cases and articles in languages other than English were excluded. Pre-clinical and ex vivo studies, long PHILOS, shaft fractures, pathologic fractures, fixation with augmentation, and review articles were also criteria for exclusion.

### 2.2. Data Extraction

Two independent reviewers (LMO and PF) screened all the titles and abstracts. After this first screening, the articles that met the inclusion criteria were screened for full-text eligibility and were excluded if they met one of the exclusion criteria. In case of disagreement between the two reviewers, a third reviewer (JA) was consulted. An electronic table for data extraction was created prior to the study using Excel (Microsoft). The following data were extracted: title, first author, year of publication, journal, type of study, population characteristics, follow-up, type of fracture, functional outcomes, surgical technique, complications, reinterventions, and plate removal reasons. Plate removal carried out at the patient’s will without giving an explanation was not included in the complication count.

The Downs and Black’s “Checklist for Measuring Quality” was used to evaluate the risk of bias [13]; it is easy to use and provides a numeric score out of a possible 32 points. It contains 27 ‘yes’-or-’no’ questions across five sections. The five sections include questions about the overall quality of the study (10 items), the ability to generalize findings of the study (3 items), the study bias (7 items), the confounding and selection bias (6 items), and the power of the study (1 item). Assessment of risk of bias and quality of evidence was completed independently for all outcomes by two authors and a third author solved any possible discrepancy.

### 2.3. Statistical Analysis

The Mantel–Haenszel method was used to provide pooled rates across the studies. A statistical test for heterogeneity was first conducted with the Cochran Q statistic and I2 metric and was considered the presence of significant heterogeneity with I2 values ≥ 25%. When no heterogeneity was found with I2 < 25%, a fixed-effect model was used to estimate the pooled rates and 95% C.I.s. Otherwise, a random-effect model was applied, and an I2 metric was evaluated for the random effect to check the correction of heterogeneity. The study’ rate confidence intervals were carried out using the continuity-corrected Wilson interval [14]. The statistical significance of the difference between groups was based on the z statistics. All statistical analysis was carried out with Microsoft Excel 2010.

## 3. Results

### 3.1. Details of the Included Studies

**Figure 1:**
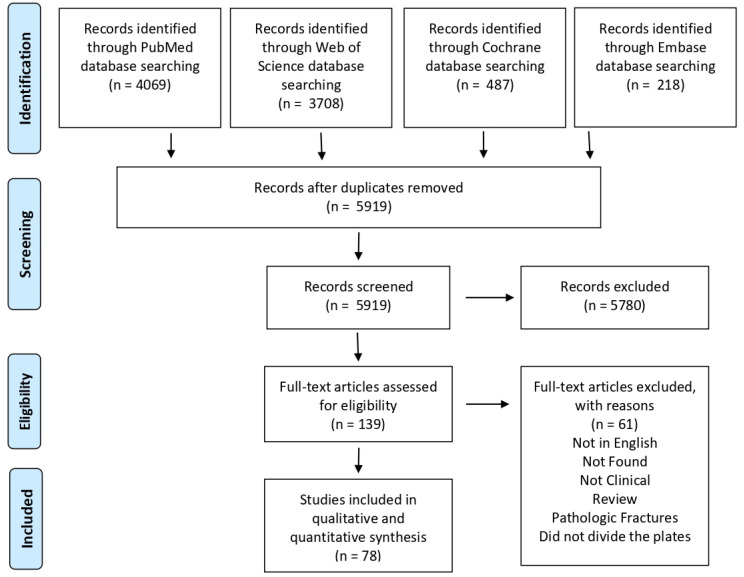
P.R.I.S.M.A. (Preferred Reporting Items for Systematic Meta-Analyses) flowchart of the study selection process. Assessment of risk of bias and quality of evidence.

A total of 8482 articles were retrieved; after the removal of duplicates, screening on the titles, abstracts, and full-texts, 78 articles were included in the systematic review (Figure 1). A total of 4200 patients (65.6% females) with 4202 fractures were included; the mean age was 60.3 years, and the mean follow-up was 19.9 months. Seventy studies reported the fracture type: 992 were two-part, 1446 three-part, and 888 four-part according to the Neer classification [15]. Moreover, 13 articles (886 fractures) included only patients older than 55 years. The standard deltopectoral (DP) approach was used in 60 studies (2966 patients) and the delto-split (DS) approach was used in 16 studies (671 patients). Among these, seven studies (547 patients) used both approaches; in addition, nine studies (563 patients) did not report the approach used (Table 1 for further details).

### 3.2. Complications and Reinterventions

One study did not report the number of complications. In the remaining 77 studies, 1229 complications were described. Fifty-seven studies (3187 patients) reported the complication rate, which was 23.8%.

**Figure 2:**
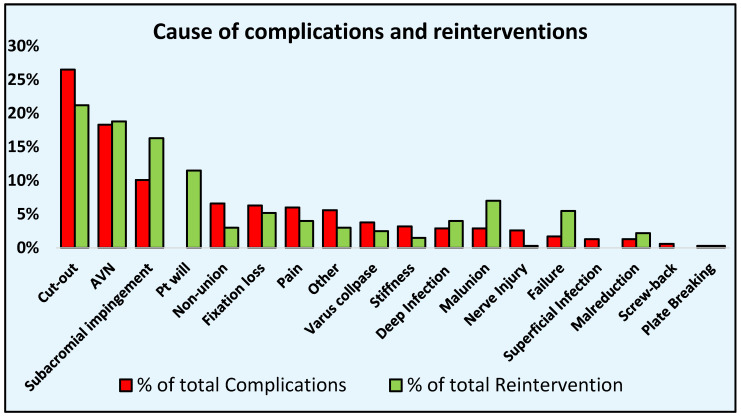
Complications and reinterventions causes; data reported as percentages of the total complications and reinterventions.

The most frequent complication was screw perforation into the joint/screw cut-out in 306 patients, with a rate of 4.1% (95% C.I. 3.2–5.1%) representing 25.7% of all complications, followed by AVN of the humeral head in 214 patients with a rate of 3.1% (95% C.I. 2.4–3.9%), representing 17.9% of all complications. The third most common complication was subacromial impingement in 121patients with a rate of 1.5% (95% C.I. 1.1–2.0%), accounting for 10.1% of all complications (Table 2) (Figure 2).

Twenty-three studies (1290 patients) reported the type of fracture in which the complication occurred, described using the Neer classification. For Neer type 4 fractures the complication rate was 38.8%, while in Neer type 3 it was 5.8% and in Neer type 2 it was 8.9%.

A total of 59 studies (3210 patients) reported the number of reinterventions performed after PHILOS plating. There was a total of 514 reinterventions on 441 patients, and the reintervention rate was 10.5% (95% C.I. 8.3–12.8%). The most common cause of reintervention was screw cut-out, followed by AVN of the humeral head and subacromial impingement (Table 3).

A total of 46 studies reported both the type of complications and the reason for reinterventions: the complications with the highest probability of reintervention was plate break (100% of the affected patients), followed by malreduction in 87.5% of the affected patients, subacromial impingement (73.6%), and loss of fixation (56.5%) (Table 4).

### 3.3. Complications and Reinterventions in Deltopectoral and Delto-Split Approaches

The studies in which the DP approach was used reported 710 complications, for a complication rate of 23.8% (95% C.I. 19.9–27.7%) (Table 4). The most common complications were screw cut-out (6.8%) and AVN of the humeral head (5.2%). These two complications accounted for 44% of the total complications. The rate of complications in the DS approach was 17.5% (95% C.I. 12.6–22.3%) (137 complications) (Table 5). Furthermore, for this approach screw cut-out and AVN of the humeral head were the two most common complications, accounting for 39.2% of all complications.

To better comprehend the relationship between surgical approach and complications for each fracture type, these two groups were divided into subgroups according to the fracture type. In the Neer 2 group, the complication rate for the DP approach was 10.1% and for the DS approach was 8.5% (95% C.I. 4.5–15.6% vs. 95% C.I. 2.5–14.6%, n.s.). In the Neer type 3 group, the complication rate for the DP approach was 13.5% and for the DS approach was 16.2% (95% C.I. 7.8–19.1% vs. 95% C.I. 2.5–29.9%, n.s.). In the Neer type 4 group, the complication rate for the DP approach was 24.1% and for the DS approach was 25.6% (95% C.I. 16.7–31.5% vs. 0.0–51.1%, n.s.). No differences between the two approaches were seen for any of the abovementioned fracture types (n.s.) (Table 6).

Regarding reinterventions, 45 studies (2301 patients) where the DP approach was used reported 291 reinterventions, for a rate of 8.6% (95% C.I. 6.5–10.7%) (Table 5), whereas in the DS approach the reintervention rate was 10.4% (95% C.I. 5.9–14.8%) (10 studies, 398 patients) (n.s.) (Table 4). The most common reasons for reintervention in both approaches were screw cut-out, AVN of the humeral head, and subacromial impingement.

### 3.4. Complications and Reinterventions in Patients over 55 Years Old

In the 13 articles including only patients over 55 years old, the complication rate was 29.5% (95% C.I. 17.6–41.4%) (347 complications) (Table 4). The most common complications included screw cut-out (35.4% of the complications) and AVN of the humeral head (16.1% of the complications). Moreover, 216 patients underwent reintervention (19.0%, 95% C.I. 9.9–28.1%) with screw cut-out being the main cause (Table 4).

### 3.5. Functional Outcome

Regarding functional outcomes, the two most used scores in the retrieved papers were the Constant and Murley Score (CMS) [16] and the Disabilities of Arm, Shoulder and Hand (DASH) score [17]. The CMS is divided into four subscales: pain (15 points), activities of daily living (20 points), strength (25 points), and range of motion (40 points); the higher the score, the higher the quality of the function, for a maximum score of 100 points. The DASH score is a 30-item, self-report questionnaire designed to measure physical function and symptoms in patients with several musculoskeletal disorders of the upper limb. It is composed of two parts: the disability/symptom section and the optional sport/music or work section. The overall DASH score ranges from 0 to 100, with 0 being the best possible score. The CMS at ≥ 12 months was reported by 39 articles, the mean was 70.8 points (95% C.I. 66.7–74.9 points). According to the CMS, functional outcomes were excellent in 3 studies (7.7%), good in 21 (53.8%), moderate in 13 studies (33.4%), and poor in 2 studies (5.1%). The DASH score at ≥12 months was reported by 16 articles, and the mean was 20.5 points (95% C.I. 16.6–24.3 points).

### 3.6. Risk of Bias

**Figure 3:**
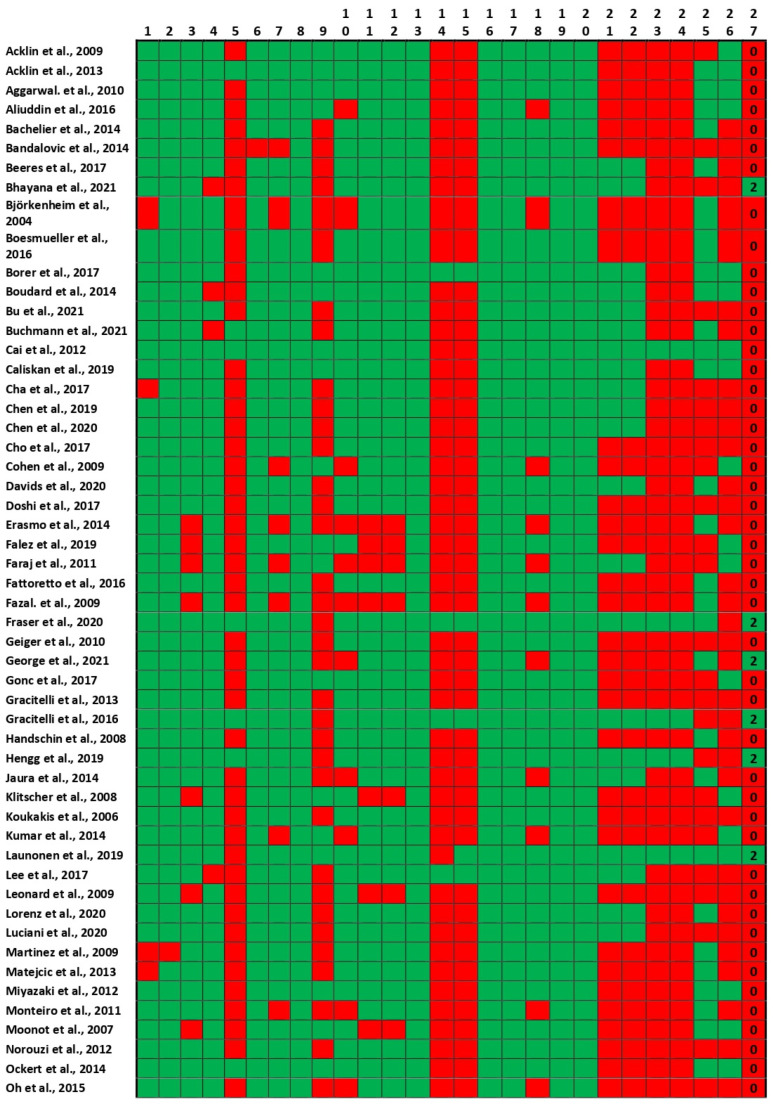

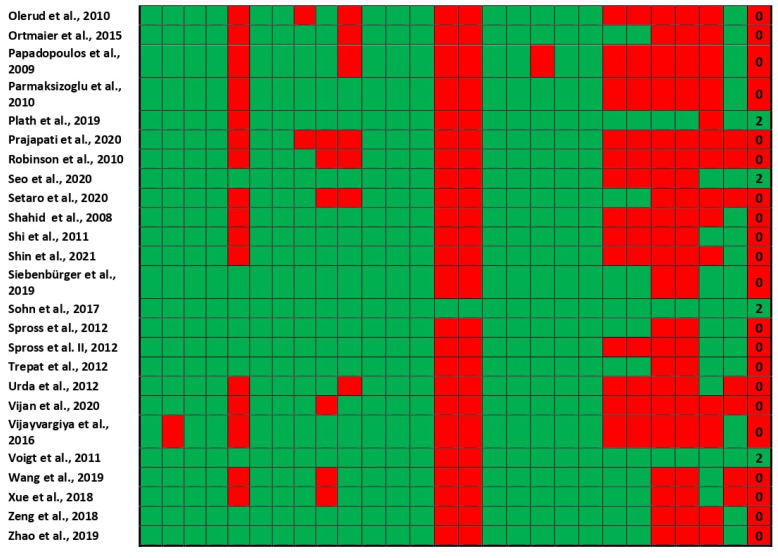
Downs and Black’s tool for assessing the risk of bias. For the explanation of each column question, see Appendix A.

The Downs and Black’s tools for assessing the risk of bias give each study an excellent ranking for scores ≥26, good for scores from 20 to 25, fair for scores between 15 and 19, and poor for scores ≤14 points. According to these criteria, 8 of the included studies were classified poor, 49 fair, 16 good, and 5 excellent (Figure 3). Mostly, the factors reducing the quality of the studies were the absence of confounders and blinding attempts and the low statistical power of some studies.

## 4. Discussion

The main finding of the present study was that proximal humerus synthesis with a PHILOS plate has a high complication rate of 23.8% and a reintervention rate of 10.5% and that these values increase up to 29.5% and 19.0%, respectively, in the over-55 population. The most frequent complication in both population groups was screw cut-out, followed by AVN of the humeral head and subacromial impingement.

Nowadays, the PHILOS plate is the most used method of PHFs fixation. Still, despite PHILOS’s large use, the current literature lacks proper investigation of the complication and reintervention rate of this surgical approach. Regarding complication rate in the PHILOS-treated population, previous reviews provided discording data: old review attempts of Sproul et al. [18] in 2010 and Kavuri et al. [19] in 2018 reported a complication rate of 32.6% and 32.8%, respectively, while more recent studies showed a wide range of complications going from 12.0% to 43.0% [18,20,21,22,23,24]. Previous reviews also presented important limitations including mixed populations treated with various plate designs. On the opposite, the present systematic review and meta-analysis present data focused on patients treated with PHILOS plate. Thus, this data helps clarify the actual prevalence of complications of the PHILOS plate through an updated and comprehensive synthesis of the literature. In this population, the complication rate was 23.8%. This may seem lower than other reports. For example, Barlow et al. reported a 34% failure rate [25]. However, it is important to consider the inclusion criteria of different studies, as in the study of Barlow et al. the focus was on patients older than 65 years, besides including different plates, which by themselves may entail different results and therefore weight on the conclusions driven. In this meta-analysis, specifically focused on the PHILOS plate, further analysis was performed to better comprehend the relationship between complication rate and age by considering only studies describing patients over 55 years old: in this population, the complication rate raised to 29.5%. This increase in the complication rate is not surprising, because this age range was described to have a 2.6 times higher risk of osteoporosis than the younger one [1,26,27,28,29,30,31,32,33,34,35,36,37,38,39].

The most frequent complication documented in the present study was screw cut-out, with a rate of 4.1%. Moreover, the previous reviews underlined this as the most frequent complication, with Sproul et al. [18] reporting a rate of 7.5%, and Kavuri et al. [19] of 9.5% in these older literature analyses. In addition, this systematic review and meta-analysis was also able to underline that patients with this type of complication have a 35.6% chance of reintervention. Given the prevalence and dangerous consequences of screw cut-out, it would have been important to further analyze this complication by dividing it into primary and secondary. Primary penetration is caused by an intraoperative surgical error, thus being preventable. This occurs when a too-long screw is inserted, and subsequently, it penetrates the cortical bone to the glenohumeral joint. On the other hand, secondary penetration of the screw into the joint occurs later and can be caused as a result of AVN, varus collapse, or failed fixation. Unfortunately, this subanalysis was not feasible since the available literature is lacking on this aspect and almost no study reported the prevalence of the two types of screw cut-out. Future clinical trials should specifically take into consideration this problem, as it could help to improve patient treatment and clinical outcomes.

AVN of the humeral head is the most dangerous complication and one of the greatest concerns for the surgeon because it necessarily implies a reintervention. It can develop within 5 years from the injury; thus, since only long-term observational studies can detect the true rate, short-term studies could have even underestimated the already high occurrence of this severe complication. AVN can be either mildly paucisymptomatic or painful, and can lead to a decreased range of motion, secondary screw perforation, and, after many years, osteoarthritis of the glenohumeral joint. The risk of developing osteonecrosis depends mainly on the complexity of the fracture, with Neer type 4 being the most susceptible one, and from the surgical approach. In this study, the rate of AVN was 3.1%. This data is different from the previous review by Sproul et al. [18], where the rate was 10.8%, but it is aligned with the more recent review by Kavuri et al. [19], where it was 4.4%. The probability of reintervention for this type of patient is 35.9%. The decrease of the AVN rate in the reviews over time may be due to different factors, such as an increased propensity in the last years to perform reverse total shoulder arthroplasties or hemi arthroplasties in patients with some severe types (e.g., loss of the medial hinge integrity or loss of the dorsomedial metaphyseal support), and the progressive use of the DS instead of the DP approach [19,40].

Finally, in this study subacromial impingement was the third most frequent complication, with a rate of 1.5%. Sproul et al. [18] reported a rate of 4.8%, while Kavuri et al. [19] of 5.0%, but their results were based on less included studies and not only on PHILOS plate, thus making the current results more reliable and comprehensive. This complication is the consequence of poor intraoperative plate placement or humeral head collapse and causes pain, rotator cuff tendons damage, and osteoarthritis development. The present analysis reports an overall low incidence of this complication for the PHILOS plate, especially when comparing it to the data referred to other plates [18,19,41] or intramedullary nailing [42]. However, patients with this type of complication have a 76% probability of reintervention, which is double the rate of the previous two complications, and consequently, as the third most frequent complication, this data is even more significant.

In addition to the analysis of complications, another important indicator of a successful operation is the reintervention rate, because of the consequences that it implies: increased discomfort for the patient, exposure to another surgical session and the inherent risks, hospitalization, etc., as underlined by Ockert et al. [34], who reported that patients who had a revision had an improvement in shoulder function, but this remained lower than the one of non-reoperated patients. Overall, the reintervention rate in this study was 10.5%, which is aligned to the recent work of Kavuri et al. [19], reporting a rate of 13.8%. Moreover, the current work underlines an increase in the reintervention rate up to 19.0% when considering only patients over 55 years. This increase can also be seen in the study of Luciani et al. [43], which considered only patients over 65 years, and described a complication rate of 34.6%, also confirming previous literature findings [44,45]. In this analysis, the main cause of reintervention was screw cut-out, with a rate of 21.2%. This complication was reported to be the main cause of reintervention also by Sproul et al. [18] and by other authors [29,35,46]. The second cause of reintervention was AVN, which had a different prevalence among the DP and DS surgical approaches: in the first approach, AVN caused 23.7% of all reinterventions, whereas in the DS approach only 7.7%.

The best surgical approach to address PHFs is an open debate among shoulder surgeons, with the DP being the most common one and the DS being the less used approach [47]. The proximal humerus has rich and fragile vascularity, therefore attention must be paid when performing the DP approach, as an inaccurate and inappropriate surgical exposure during plating increases the risk of osteonecrosis due to a possible injury of the anterior circumflex humeral artery. In contrast, the DS approach reduces soft tissue dissection of the injured region and promotes biological healing at the fracture site, while simultaneously reducing the risk of osteonecrosis. On the other hand, the DS approach implies an increased risk of damage to the axillary nerve, since the insertion and fixation of the plate are in its proximity [48]. Smith et al. [49] reported how this problem can be overcome by using a six-hole PHILOS plate and inserting the screw in the proximal hole and not in the inferior medial oblique hole. Previous literature [50,51,52], as also reported by the studies of Li et al. [53] and Sohn et al. [47], was not able to find any statistical difference between the DP and DS approaches in terms of complications.

In this systematic review, the overall complication rate in the DP approach was 23.8%, and in the DS approach, it was 17.5%. This result brings a very important addition to the literature, although no statistically significant difference was found, there is a trend difference in the complication rate between the two approaches (*p* = 0.054). However, besides the different surgical approaches, this result can also be explained by the fact that the DS approach was mainly used for Neer type 2 and 3 fractures, which are easier to treat and carry a lower risk of complications when compared to Neer type 4 fractures [54,55,56]. In fact, the subanalysis showed that when the DS approach was used in Neer type 4 fractures, it carried a higher risk of complications, probably due to the original trauma itself, as well as the need of these complex fractures for a more dissection of the soft tissues to have better exposure of the fracture site. To this regard, Sohn et al. [47] and Shin et al. [57] recommend a DP approach in Neer type 4 fractures, as a DS approach would cause inadequate fracture alignment, resulting in lower functional outcomes and less patient satisfaction, as largely confirmed by the literature [58,59,60,61,62].

Up to now, there is still a lack of clear guidelines on the use of operative or conservative treatments: operative treatment with the PHILOS plate for young patients is a well-established procedure, whereas for elderly patients with different degrees of osteoporosis and displaced fractures the indications are still not clear, although operative treatment is steadily increasing [63,64]. Given the debated topic, many authors attempted to assess the real benefits of the plate over conservative treatment in the over 60 population. Four recent RCTs compared the two different treatments, showing that no clinical relevance exists to support the surgical approach for any type of Neer fracture [31,65,66,67], despite the fact that the surgical approach leads to better bone alignment. This result is very important since PHFs are one of the main fractures in the elderly population and consequently the choice of a conservative approach could imply a lower risk of surgically related complications, as underlined by the fact that these RCTs consistently reported a higher complication rate for the locking plate group than for the non-operative treatment group, although in no study a statically significant correlation was found [31,65,66,67]. These studies reported that subacromial impingement was the main complication in conservatively treated PHFs, but the incidence was lower than the one reported for both locking plate and PHILOS plate treatments. On the other hand, Boons et al. [64], in their systematic review, reported malunion as the main complication in conservative treatment of PHF Neer 4, while the current study found that less than 1% of PHILOS patients can expect this complication. Finally, nowadays neither approach prevails over the other, and both have their pros and cons. Thus, this meta-analysis is of clinical relevance, as it adds important data useful to shed more light on the potential and limitations of the PHILOS approach, which should be considered when managing PHFs patients.

Because of the high rate of complications encountered in PHFs fixation with PHILOS plate, it is important to also assess the clinical outcomes of patients, by using worldwide validated functional scores. In this meta-analysis, the two most used scores in the retrieved paper were CMS and DASH: the first had a mean of 70.8 points and 60% of the included patients achieved a good or better CMS level according to the author’s conclusions. This finding is in agreement with the most recent literature, as reported in the studies of Launonen et al. [31] and Olerud et al. [65] which showed an average CMS value similar to that found in this review. Furthermore, in recent years, following the increasing use of locking plates, several authors published studies comparing locking plates for PHFs with conservative strategies, but none of them found better functional outcomes for the surgical treatment, apart from Olerud et al. [65] who, in their RCT, showed that patients treated with locking plate had a faster clinical improvement and better quality of life than those treated conservatively, although without a statistically significant difference and with a 30% risk of additional surgery. This systematic review and meta-analysis builds upon the data of the previous literature on the complications of locking plates further implementing these data, by analyzing a larger number of patients and studies with a specific focus on the PHILOS plate. However, some limitations are still present [18,41]. First of all, the lack of comparative and randomized studies. Second, no study reported homogenous groups of patients <55 years old, thus impairing a comparative analysis between different age ranges. However, the principal strength of this study is the large number of articles and patients included, the clarity of inclusion and exclusion criteria, and the analysis of studies using exclusively PHILOS plates to treat PHFs. Moreover, only studies with a minimum follow-up of 12 months were included, giving the possibility to consider the surgical results stabilized. Thus, overall, this comprehensive review of the complications documented after PHILOS synthesis offers important indications for shoulder and trauma surgeons and suggests the need to further improve the treatment of PHFs to reduce complications and reinterventions.

## 5. Conclusions

Proximal humerus synthesis with a PHILOS plate has a complication rate of 23.8%, with a reintervention rate of 10.5%, and these values increase up to 29.0% and 19.0%, respectively, in the over-55 population. The most frequent complication in both the overall and the older population groups was screw cut-out, followed by AVN of the humeral head and subacromial impingement. These results of the PHILOS plate will have to be further investigated to better understand both the type of patient and fracture that is more at risk of complications and reintervention and to compare pros and cons with respect to the other solutions to manage patients affected by PHFs.

## Figures and Tables

**Table 1:** Details of the included studies; Pt. patients, M male, F female, N Neer Classification, DS delto-split, DP delto-pectoral.

First Author; Year	Country	Comparative	Pt. (M−F)	Age	Neer Classification			Mean Follow-Up	Surgical Approach
					N2	N3	N4		
Acklin et al., 2009	Switzerland	NO	29 (9−20)	64	N/A	N/A	N/A	12	DS
Acklin et al., 2013	Switzerland	NO	97 (N/A)	62	N/A	N/A	N/A	18	DS
Aggarwal et al., 2010	India	NO	47 (27−20)	58.5	11	22	14	21.5	DP
Aliuddin et al., 2016	Pakistan	NO	20 (12−8)	40	4	10	6	6	DP
Bachelier et al., 2014	Germany	NO	50 (20−30)	62.7	15	18	17	12	DS
Bandalovic et al., 2014	Croatia	NO	67 (N/A)	N/A	N/A	N/A	N/A	14.7	DP/DS
Beeres et al., 2017	Switzerland	YES	282 (85−197)	64	58	153	74	12.3	DP/DS
Bhayana et al., 2021	India	YES	84 (45−39)	45	0	40	44	23	DP/DS
Björkenheim et al., 2004	Finland	NO	72 (28−44)	67	38	22	12	12	DP
Boesmueller et al., 2016	Austria	NO	154 (61−93)	55.8	41	71	42	15.5	DP
Borer et al., 2017	Switzerland	YES	62 (16−46)	64	18	4	10	51	DP/DS
Boudard et al., 2014	France	YES	33 (19−14)	49.6	0	21	12	24.7	DP
Bu et al., 2021	China	YES	48 (17−31)	66.3	28	13	7	15.6	DP
Buchmann et al., 2021	Switzerland	YES	198 (75−123)	64.3	N/A	N/A	N/A	12	DP/DS
Cai et al., 2012	China	YES	12 (1−11)	72.4	0	0	12	24	DP
Caliskan et al., 2019	Turkey	YES	45 (18−27)	53.2	11	21	13	25	DS
Cha et al., 2017	South Korea	YES	32 (8−24)	67.8	8	21	3	15	DP
Chen et al., 2019	China	YES	112 (37−75)	64.29	52	60	0	15	DP
Chen et al., 2020	Taiwan	YES	35 (13−22)	56.1	12	17	6	12	DP
Cho et al., 2017	South Korea	NO	39 (12−27)	59	14	22	3	45	DP
Cohen et al., 2009	Brazil	NO	26 (12−14)	57	7	10	7	12	DP
Davids et al., 2020	USA	YES	75 (N/A)	59.9	40	35	0	17.6	DP
Doshi et al., 2017	India	NO	53 (24−29)	54.3	19	17	11	12	DP
Erasmo et al., 2014	Italy	NO	81 (39−42)	56	7	40	35	32	DP
Falez et al., 2019	Italy	NO	76 (26−50)	68.5	3	35	38	12	DS
Faraj et al., 2011	Netherlands	YES	37 (N/A)	N/A	N/A	N/A	N/A	29	DS
Fattoretto et al., 2016	Italy	NO	55 (17−38)	63.4	0	16	39	21.5	DP/DS
Fazal et al., 2009	UK	NO	27 (6−21)	56	13	12	2	13	DP
Fraser et al., 2020	Norway	YES	60 (8−52)	74.7	0	29	31	24	DP
Geiger et al., 2010	Germany	NO	28 (8−20)	60.7	8	12	8	25.2	DP
George et al., 2021	India	NO	35 (25−10)	52	12	21	14	6	DP
Gonc et al., 2017	Turkey	NO	31 (12−19)	58.4	4	14	13	12	DS
Gracitelli et al., 2013	Brazil	NO	40 (12−28)	61.8	16	22	2	12	DP
Gracitelli et al., 2016	Brazil	YES	33 (8−25)	66.4	16	17	0	12	DP
Handschin et al., 2008	Switzerland	NO	31 (11−20)	62	8	13	10	19	DP
Hengg et al., 2019	Austria	YES	34 (5−29)	76	5	17	12	12	DP
Jaura et al., 2014	India	YES	30 (20−10)	65	12	14	4	12	DP
Klitscher et al., 2008	Germany	NO	30 (11−19)	59	2	16	12	16.4	DP
Koukakis et al., 2006	Greece	NO	20 (8−12)	61.7	5	11	4	16.2	DP
Kumar et al., 2014	India	NO	51 (35−16)	38	8	15	23	30	DP
Launonen et al., 2019	UK	YES	44 (3−41)	82	44	0	0	24	N/A
Lee et al., 2017	South Korea	YES	31 (11−20)	58.6	31	0	0	21	N/A
Leonard et al., 2009	Ireland	NO	32 (9−23)	61.6	N/A	N/A	N/A	14	DP
Lorenz et al., 2020	Austria	YES	31 (N/A)	59	0	12	19	12	DP
LuC.I.ani et al., 2020	Italy	YES	26 (3−23)	73	0	9	15	40	DP
Martinez et al., 2009	Spain	NO	58 (31−27)	61	0	33	25	15	DP
MatejC.I.c et al., 2013	Croatia	NO	59 (9−50)	70.5	0	32	27	19	DP
Miyazaki et al., 2012	Brazil	NO	56 (19−37)	62	13	28	8	12	DP
Monteiro et al., 2011	Brazil	NO	33 (14−19)	57	17	13	4	24	DP
Moonot et al., 2007	UK	NO	32 (9−23)	59.9	0	20	12	11	DP
Norouzi et al., 2012	Iran	NO	37 (27−10)	50.1	13	20	4	12	N/A
Ockert et al., 2014	Germany	NO	43 (12−31)	58.2	N/A	N/A	N/A	120	DP
Oh et al., 2015	Germany	NO	26 (6−20)	67	0	17	9	20.1	DS
Olerud et al., 2010	Sweden	NO	50 (10−40)	75	50	0	0	N/A	DP
Ortmaier et al., 2015	Austria	YES	30 (13−17)	31.3	0	10	20	38.4	N/A
Papadopoulos et al., 2009	Greece	NO	29 (12−17)	62.3	0	22	7	17.9	DP
Parmaksizoglu et al., 2010	Turkey	NO	32 (10−22)	63	0	12	20	25	DP
Plath et al., 2019	Germany	YES	32 (7−25)	77.1	4	24	4	12.8	DP/DS
Prajapati et al., 2020	India	YES	20 (5−15)	41	N/A	N/A	N/A	12	DP/DS
Robinson et al., 2010	Scotland	NO	47 (21−26)	57	27	12	8	24	DS
Seo et al., 2020	South Korea	NO	27 (12−15)	53	5	14	8	15.9	DP
Setaro et al., 2020	Italy	YES	64 (N/A)	61.5	37	27	0	48	DP
Shahid et al., 2008	UK	NO	41 (9−32)	N/A	11	11	19	12	DP
Shi et al., 2011	China	NO	43 (15−28)	68.7	10	21	12	12	DP
Shin et al., 2021	South Korea	NO	56 (12−44)	74.3	21	27	8	15.4	DP
Siebenbürger et al., 2019	Germany	YES	55 (12−43)	76.6	20	22	13	24	DP
Sohn et al., 2017	South Korea	YES	90 (N/A)	61.8	35	44	11	14.7	DP/DS
Spross et al., 2012	Switzerland	YES	22 (4−18)	75	N/A	N/A	N/A	30	DP
Spross et al., 2012	Switzerland	NO	294 (71−223)	72.9	N/A	N/A	N/A	12	DP
Trepat et al., 2012	Spain	YES	11 (3−8)	68.3	11	0	0	6	DP
Urda et al., 2012	Spain	NO	15 (3−12)	71	15	0	0	40.67	DP
Vijan et al., 2020	India	YES	15 (N/A)	52.3	7	6	2	12	N/A
Vijayvargiya et al., 2016	India	NO	26 (19−7)	46	5	12	9	12	DS
Voigt et al., 2011	Germany	YES	31 (N/A)	72	0	27	4	12	DP
Wang et al., 2019	China	YES	46 (13−33)	72.5	0	0	46	19	DP
Xue et al., 2018	China	YES	43 (N/A)	57	43	0	0	N/A	DS
Zeng et al., 2018	China	YES	181 (64−117)	57.4	78	75	28	12	DP
Zhao et al., 2019	China	YES	21 (12−9)	69	0	15	6	12	DP

**Table 2:** Complication type; n° and rate of complications.

Complication Type	N° of Complications	Complication Rate	% of the Total
Total complications	1229	29.1	100%
Cut-out	313	7.5	25.7%
AVN	215	5.1	17.6%
Subacromial Impingement	121	2.9	9.9%
Non-union	79	1.9	6.4%
Fixation Loss	76	1.8	6.3%
Pain	70	1.6	5.7%
Others	65	1.5	5.3%
Varus collapse	45	1.1	3.6%
Stiffness	41	0.9	3.1%
Deep Infection	34	0.8	2.8%
Malunion	36	0.8	2.9%
Nerve Injury	30	0.7	2.4%
Failure	20	0.5	1.6%
Superficial Infection	15	0.4	1.2%
Malreduction	14	0.3	1.2%
Screw-back	7	0.2	0.6%
Plate Breaking	4	0	0.3%
Unknown	42	1	3.4%

**Table 3:** Reasons for reintervention; n° and rate of reinterventions.

Reintervention Reason	N ° of Reinterventions	Reintervention Rate	% of the Total
Total Reintervention	514	16	100
Cut-out	69	2.2	13.4
AVN	61	1.9	11.9
Subacromial Impingement	53	1.7	10.3
Pt will	37	1.2	7.2
Failure	18	0.6	3.5
Loss Fixation	17	0.5	3.3
Deep Infection	13	0.4	2.5
Non-Union	10	0.3	2.0
Pain	8	0.2	1.6
Varus Collapse	8	0.2	1.6
Other	8	0.2	1.6
Mal Reduction	7	0.2	1.5
Stiffness	5	0.2	0.9
Plate Discomfort	5	0.2	0.9
Malunion	2	0	0.4
Frozen Shoulder	2	0	0.4
Screw Back	2	0	0.4
Nerve Injury	1	0	0.2
Plate Break	1	0	0.2
Unknown	187	5.8	36.2

**Table 4:** Probability of reintervention for type of complication.

Type of Complications	% of Complication Causing Reintervention
Cut-out	32.5
AVN	35.9
Subacromial impingement	73.6
Pain	8.4
Loss fixation	56.5
Non-union	26.3
Other	25.0
Varus collapse	25.8
Deep infection	33.3
Malunion	8.0
Stiffness	27.8
Nerve injury	10
Malreduction	87.5
Superficial infection	0.0
Screw-back	0.0
Plate breaking	100

**Table 5:** Details Pt with Complications and Reintervention.

	Pt. with Complications	N° of Complications	Complications Rate	Pt. with Reintervention	N° of Reintervention	Reintervention Rate
>55 years	836	347	29.5	761	216	19.0
DP group	2910	810	23.8	2301	291	8.6
DS group	657	137	17.5	398	53	10.4
Pt. tot	4200	1229	23.8	3210	514	10.5

**Table 6:** Complication rate normalized for the type of fracture and surgical approach.

Complication Rate	Neer Type 2	Neer Type 3	Neer Type 4
DP group	10.1 ± 2.8% (95% C.I. 4.5–15.6)	13.5 ± 2.9% (95% C.I. 7.8–19.1)	24.1 ± 3.8% (95% C.I. 16.7–31.5)
DS group	8.5 ± 3.0% (95% C.I. 2.5–14.6)	16.2 ± 7.0% (95% C.I. 2.5–29.9)	25.6 ± 7.0% (95% C.I. 0.0–51.1)
*p* Value	0.37	0.37	0.39

## Data Availability

The data presented in this study are available on request from the corresponding author.
